# Influence of insecticide resistance on the biting and resting preferences of malaria vectors in the Gambia

**DOI:** 10.1371/journal.pone.0241023

**Published:** 2021-06-24

**Authors:** Majidah Hamid-Adiamoh, Davis Nwakanma, Benoit Sessinou Assogba, Mamadou Ousmane Ndiath, Umberto D’Alessandro, Yaw A. Afrane, Alfred Amambua-Ngwa

**Affiliations:** 1 West African Centre for Cell Biology of Infectious Pathogens (WACCBIP) and Department of Biochemistry, Cell and Molecular, University of Ghana, Legon, Accra, Ghana; 2 Medical Research Council Unit, The Gambia at The London School of Hygiene & Tropical Medicine, Banjul, The Gambia; 3 Department of Medical Microbiology, University of Ghana Medical School, University of Ghana, Accra, Ghana; Kansas State University, UNITED STATES

## Abstract

**Background:**

The scale-up of indoor residual spraying and long-lasting insecticidal nets, together with other interventions have considerably reduced the malaria burden in The Gambia. This study examined the biting and resting preferences of the local insecticide-resistant vector populations few years following scale-up of anti-vector interventions.

**Method:**

Indoor and outdoor-resting *Anopheles gambiae* mosquitoes were collected between July and October 2019 from ten villages in five regions in The Gambia using pyrethrum spray collection (indoor) and prokopack aspirator from pit traps (outdoor). Polymerase chain reaction assays were performed to identify molecular species, insecticide resistance mutations, *Plasmodium* infection rate and host blood meal.

**Results:**

A total of 844 mosquitoes were collected both indoors (421, 49.9%) and outdoors (423, 50.1%). Four main vector species were identified, including *An*. *arabiensis* (indoor: 15%, outdoor: 26%); *An*. *coluzzii* (indoor: 19%, outdoor: 6%), *An*. *gambiae s*.*s*. (indoor: 11%, outdoor: 16%), *An*. *melas* (indoor: 2%, outdoor: 0.1%) and hybrids of *An*. *coluzzii-An*. *gambiae s*.*s* (indoors: 3%, outdoors: 2%). A significant preference for outdoor resting was observed in *An*. *arabiensis* (Pearson *X*^*2*^ = 22.7, df = 4, P<0.001) and for indoor resting in *An*. *coluzzii* (Pearson *X*^*2*^ = 55.0, df = 4, P<0.001). Prevalence of the voltage-gated sodium channel (*Vgsc*)*-1014S* was significantly higher in the indoor-resting (allele freq. = 0.96, 95%CI: 0.78–1, P = 0.03) than outdoor-resting (allele freq. = 0.82, 95%CI: 0.76–0.87) *An*. *arabiensis* population. For *An*. *coluzzii*, the prevalence of most mutation markers was higher in the outdoor (allele freq. = 0.92, 95%CI: 0.81–0.98) than indoor-resting (allele freq. = 0.78, 95%CI: 0.56–0.86) mosquitoes. However, in *An*. *gambiae s*.*s*., the prevalence of *Vgsc-1014F*, *Vgsc-1575Y* and *GSTe2-114T* was high (allele freq. = 0.96–1), but did not vary by resting location. The overall sporozoite positivity rate was 1.3% (95% CI: 0.5–2%) in mosquito populations. Indoor-resting *An*. *coluzzii* had mainly fed on human blood while indoor-resting *An*. *arabiensis* fed on animal blood.

**Conclusion:**

In this study, high levels of resistance mutations were observed that could be influencing the mosquito populations to rest indoors or outdoors. The prevalent animal-biting behaviour demonstrated in the mosquito populations suggest that larval source management could be an intervention to complement vector control in this setting.

## Introduction

Successful implementation of indoor residual spraying (IRS) and long-lasting insecticidal nets (LLINs) has hugely contributed to the malaria decline observed in sub-Saharan Africa [1]. These interventions reduce transmission by primarily limiting human contact with human-feeding (anthropophagic), indoor-feeding (endophagic) and indoor-resting (endophilic) vectors [2]. Unfortunately, these measures also induce selection for physiological and behavioral resistance in vector populations, resulting in reduced mosquito susceptibility to most of the current insecticides used for LLINs and IRS [3], as well as increased exophilic behavioral phenotypes in primarily endophilic vectors [4]. Moreover, residual transmission partly driven by high LLINs and IRS use, is maintained by vectors with physiological and behavioral resistance [5]. Therefore, studying the behavioral dynamics of vector populations during the scale up of vector control interventions will assist in determining the appropriate response to emerging behavioral changes.

Malaria burden in The Gambia has declined significantly over the last decades with vector control approaches being a major component of intervention, coordinated and implemented by The Gambia National Malaria Control Program (GNMCP). Following the World Health Organization (WHO) Global Plan for Insecticide Resistance Management (GPIRM), the GNMCP has consistently implemented rotational use of different classes of insecticides for IRS, to curtail dichlorodiphenyltrichloroethane (DDT) and deltamethrin resistance. For IRS, DDT was replaced initially by deltamethrin and bendiocarb, and since 2017 by pirimiphos-methyl (actellic 300CS) [6]. Similarly, LLINs intervention has been stable over the years and Gambia has recorded successful LLINs coverage as high as 90% [7, 8].

Despite such successes, residual transmission has become increasingly spatially heterogeneous, with its intensity increasing from western to eastern Gambia, and could have been driven by specific vector population dynamics [7]. The major vector species, namely *Anopheles arabiensis*, *An*. *coluzzii* and *An*. *gambiae sensu stricto* (*s*.*s*.) are variably distributed throughout the country. *An*. *arabiensis* is most prevalent in the eastern Gambia while *An*. *coluzzii* and *An*. *gambiae s*.*s*. inhabit the western region [9, 10]. However, *An*. *arabiensis* has been recently found throughout the country [11], indicating possible replacement due to successful control of other sibling species [12, 13]. Moreover, the population prevalence of each vector species varies by season, whereby *An*. *arabiensis* and *An*. *coluzzii* are dominant throughout the rainy season, while *An*. *gambiae s*.*s*. become rarest early in the onset of dry season [9, 10]. DDT and pyrethroid resistance has been reported at various degrees in all vectors, that continue to be highly susceptible to carbamates and organophosphates [11, 14, 15].

Host seeking and resting behavior of vectors are important metrics to evaluate the impact of control and resistance management strategies [16]. Vector behavioral adaptation, resistance selection and persistent transmission could increase during extensive scale-up of interventions, and this information can only be captured by real-time surveillance [17, 18]. Hence, national malaria control programs should actively monitor behavioral dynamics in the local vector population, to inform decisions.

In the Gambia, DDT and pyrethroid resistance is widespread and associated with residual transmission [11, 14]. However, the effect of control activities on vectors feeding and resting behavior remains unclear. The biting and resting preferences of *An*. *gambiae sensu lato* (*s*.*l*.*)* populations was investigated in The Gambia following few years of intensive vector control interventions.

## Materials and methods

### Ethical clearance

Ethical approval was obtained from The Gambia Government/Medical Research Council (MRC) Unit The Gambia at London School of Hygiene and Tropical Medicine (LSHTM) Joint Ethics Committee. The permit Number was: SCC 1586.

### *Anopheles gambiae s*.*l*. collection

**Fig 1:**
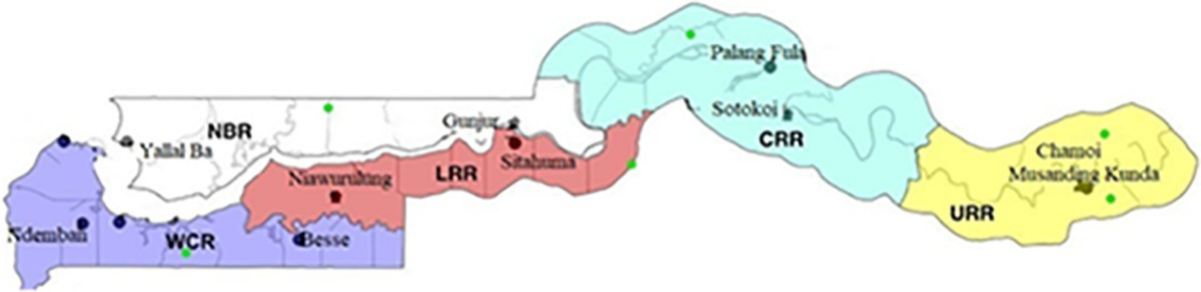
Map of The Gambia showing the study sites, comprising two villages each from the 5 administrative regions in the country.

Indoor and outdoor-resting adult mosquitoes were sampled from July to October 2019, during the malaria transmission season across five administrative regions in The Gambia, namely Central River Region (CRR), Lower River Region (LRR), North Bank Region (NBR), Upper River Region (URR) and West Coast Region (WCR) (Fig 1). WCR is a coastal area characterized by mangrove swamps. The remaining regions are mainly inland and have forest vegetation. Rice is mainly cultivated in CRR while cereals farming is common in all regions. Two villages were selected from each region and most of the villages are GNMCP surveillance sites with high LLIN and IRS coverage. Malaria transmission is highest in URR compared to other regions in The Gambia [7].

Indoor-resting mosquitoes were collected from sleeping rooms using pyrethrum spray collection (PSC). Twenty houses per village, at least 50m apart from each other, were randomly selected. In each village, collections were done for two consecutive days, with ten houses sampled per day. Outdoor-resting mosquitoes were sampled from pit shelter traps using prokopak aspirator. Three pit shelter traps that were 10m away from the selected compounds, were placed at different parts in each village. Both indoor and outdoor collections were conducted from 06.00 am to 09.00am in every collection day.

### Mosquito identification

Morphological identification of female *An*. *gambiae s*.*l*. was done using identification keys as described by Gillies & Coetzee [19]. Afterwards, mosquitoes were stored individually in 96% ethanol in 1.5ml Eppendorf tube until DNA extraction. DNA was extracted separately from abdomen and head/thoraces of individual mosquitoes using Qiagen QIAxtractor robot. Species-specific genotyping PCR to identify *An*. *arabiensis*, *An*. *melas* and *An*. *gambiae* was performed using specific primers to discriminate the species as previously done [20]. This was followed by restriction enzyme digestion to specifically identify *An*. *coluzzii*, *An*. *gambiae s*.*s*. and their hybrids (*An*. *coluzzii*-*An*. *gambiae s*.*s*.) [21].

### Insecticide resistance markers identification

Screening for molecular markers of target-site resistance to carbamates, DDT, pyrethroids and organophosphates was done on all samples using a probe-based assay (TaqMan SNP genotyping) [22]. The following markers were investigated: voltage-gated sodium (*Vgsc*)*-1014F*, *Vgsc-1014S* and *Vgsc-1575Y* associated with target-site mutation to DDT and pyrethroids [23–25]. Acetylcholine esterase (*Ace*)*-119S*, marker for carbamate and organophosphate resistance [26] and glutathione-S-transferase epsilon 2 (*Gste2*)*-114T*, involved in metabolic resistance to DDT [27] were also assayed. The TaqMan allelic discrimination assay used is a multiplex real time PCR, where primers and probes specific for each insecticide target gene were employed to discriminate susceptible (wild type) and resistant (mutant) alleles based on probe fluorescence signals [28].

## Plasmodium sporozoite detection

DNA extracted from mosquito head and thoraces was used to detect sporozoites of *Plasmodium falciparum*, *P*. *ovale*, *P*. *malariae* and *P*. *vivax* species, employing TaqMan SNP genotyping protocol [29] which enables discriminatory identification of circum-sporozoites (CSPs) of *P*. *falciparum* from *P*. *ovale*, *P*. *malariae* and *P*. *vivax* CSPs. Genomic DNA specific to each of these *Plasmodium* species were analyzed in each assay as positive controls.

### Blood meal identification

Extracted DNA from engorged mosquito abdomens were amplified using modified multiplex PCRs with specific primers that amplify cytochrome B genes of human and animal hosts including chicken, cow, dog, donkey, goat, horse and pig [30, 31].

### Statistical analyses

The proportion of each mosquito species in relation to the total number of mosquitoes captured from each region was calculated in percentage, as well as allele frequencies of indoor and outdoor-resting mosquitoes. Sporozoite positivity rate was the proportion of PCR positive mosquitoes among all mosquitoes tested. Human (HBI) and animal blood meal indices were estimated as the proportion of mosquitoes positive for human or animal hosts among those positive for all hosts. Mean differences between HBI and animal blood meal indices by vector species and resting locations were analyzed by ANOVA. Statistical analyses were done using Stata/IC 15.0 (2017 StataCorp LP).

## Results

### Anopheles species distribution and their resting behavior

**Fig 2:**
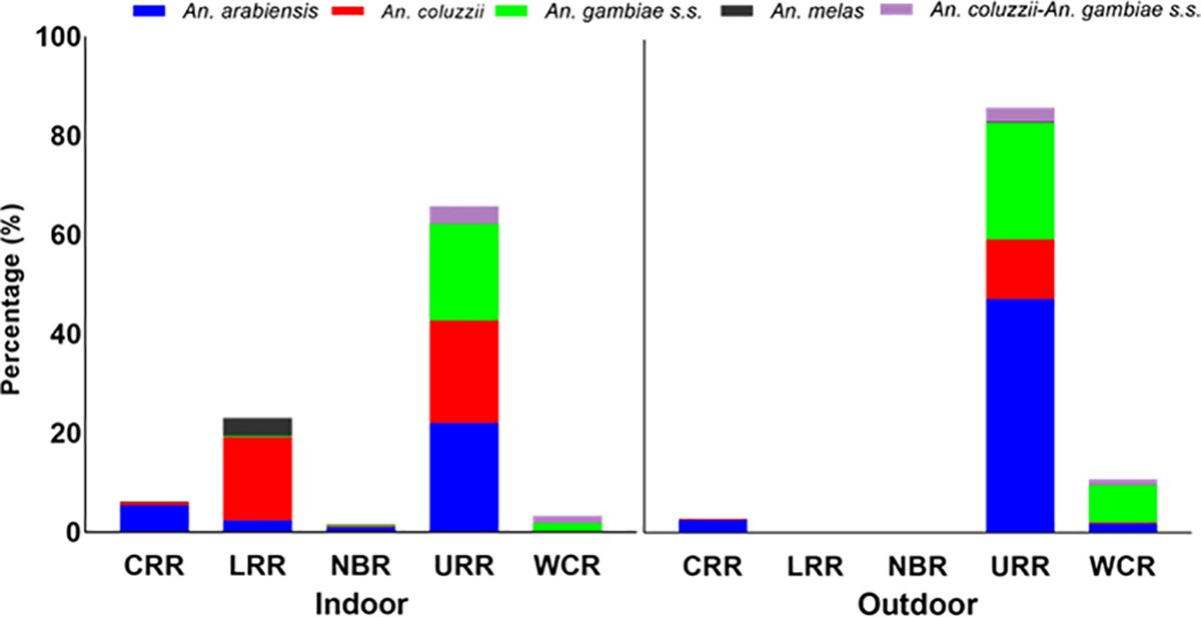
Distribution of *Anopheles gambiae s*.*l* by region as collected indoors and outdoors. *An*. *col*uzzii-An. *gambiae s*.*s*. are the hybrids of *An*. *coluzzii* and *An*. *gambiae s*.*s*. Mosquitoes were collected from 5 regions: CRR- central river region, LRR-lower river region. NBR- north bank region. URR-upper river region and WCR- West coast region.

A total of 844 *An*. *gambiae s*.*l*. mosquitoes were collected from the five regions. Four main vector species were identified, namely *An*. *arabiensis* (N = 350, 41%); *An*. *coluzzii* (N = 214, 25%), *An*. *gambiae s*.*s*. (N = 224, 27%) and *An*. *melas* (N = 17, 2%). Hybrids of *An*. *coluzzii-An*. *gambiae s*.*s*. were also detected (N = 39, 5%). Most mosquitoes were collected from URR (642, 76%), followed by LRR (97, 11%) and then the other regions (Fig 2).

Overall, the number of mosquitoes resting indoors (421, 49.9%) and outdoors (423, 50.1%) were similar. Nevertheless, the resting preference varied by species. A significantly higher proportion of *An*. *arabiensis* were found outdoor (26.1%) than indoor (15.4%) (Pearson *X*^*2*^ = 22.7, df = 4, P<0.001) while both *An*. *coluzzii* (19.1% indoor and 6.3% outdoor, Pearson *X*^*2*^ = 55.0, df = 4, P<0.001) and *An*. *melas* (1.9% indoor and 0.1% outdoor, Pearson *X*^*2*^ = 13.3, df = 4, P<0.01) preferred resting indoor. For *An*. *gambiae s*.*s*. (10.9% indoor and 15.6% outdoor, Pearson *X*^*2*^ = 7.0, df = 4, P = 0.14) and *An*. *coluzzii-An*. *gambiae s*.*s*. hybrids (2.6% indoor and 2% outdoor, Pearson *X*^*2*^ = 0.7, df = 4, P = 0.45), there was no significant difference between resting indoor and outdoor. In URR, the region with the highest malaria transmission in The Gambia, *An*. *arabiensis* was most abundant vector (45.8%, 294) (indoor: 14.5%, outdoor: 31.3%), followed by *An*. *gambiae s*.*s*. (28.4%, 182) (indoor: 12.8%, outdoor: 15.6%) and *An*. *coluzzii* (21.5%, 138) (indoor: 13.6%, outdoor: 7.9%). No *An*. *gambiae s*.*s*. was collected in CRR while *An*. *melas* was mainly found in LRR (N = 15). All mosquitoes collected from LRR and NBR were resting indoors. The hybrids of *An*. *coluzzii* and *An*. *gambiae s*.*s*. were mainly found in URR (indoor: 2.3%, outdoor: 1.9%) and WCR (indoor: 10%, outdoor: 8.3%).

### Distribution of voltage-gated sodium channel (Vgsc) mutation markers in the vectors

Vgsc point mutations associated with DDT and pyrethroid resistance were highly prevalent and detected at varying frequencies in all vector species across all regions. Overall, *An*. *arabiensis* was found resting indoors when resistance allele frequency was higher in the indoor population, whereas *An*. *coluzzii* were resting outdoors with higher outdoor resistance. No consistent resting preference was observed in *An*. *gambiae* in the presence of mutations.

*Vgsc-1014S* mutation was found predominantly in indoor-resting vector populations (Table 1). In *An*. *arabiensis*, the mutation was more frequent in the indoor-resting than outdoor-resting mosquitoes regardless of the region. V*gsc-1014S* was also the only mutation identified in *An*. *gambiae s*.*s*. and *An*. *melas* when found resting indoors.

**Table 1:** Frequencies of insecticide resistance alleles on VGSC, GST and AChE loci in *Anopheles gambiae s*.*l*. populations from all study regions.

Region	Anopheles species	*Vgsc-1014F*		*Vgsc-1014S*		*Vgsc-1575Y*		*GSTe2-114T*		*Ace1-119S*	
		Indoor	Outdoor	Indoor	Outdoor	Indoor	Outdoor	Indoor	Outdoor	Indoor	Outdoor
**URR**	***An*. *arabiensis***	0.05	0.02	0.91	0.82	0	0.004	0	0.01	0	0
		N = 5	N = 4	N = 85	N = 164		N = 1		N = 1		
	***An*. *coluzzii***	0.74	0.92	0.25	0.04	0.68	0.9	0.78	0.9	0	0
		N = 64	N = 47	N = 22	N = 2	N = 59	N = 46	N = 68	N = 46		
	***An*. *gambiae s*.*s*.**	1	0.99	0	0.01	0.96	0.98	0.98	0.99	0.05	0.04
		N = 82	N = 99		N = 1	N = 79	N = 98	N = 80	N = 99	N = 4	N = 4
	***An*. *coluzzii-An*. *gam biae s*.*s***	0.93	1	0	0	0.87	1	0.87	0	0.07	0
		N = 14	N = 12			N = 13	N = 12	N = 13		N = 1	
**LRR**	***An*. *arabiensis***	0	-	0.9	-	0	-	0.1	-	0	-
				N = 9		N = 4		N = 1			
	***An*. *coluzzii***	0.66	-	0.3	-	0	-	0	-	0	-
		N = 47		N = 21							
	***An*. *gambiae s*.*s***	0	-	1	-	0	-	0	-	0	-
				N = 1							
	***An*. *melas***	0	-	1	-	0	-	0	-	0	-
				N = 15							
**WCR**	***An*. *arabiensis***	-	0.13	-	0.88	-	0	-	0	-	0
			N = 1		N = 7						
	***An*. *coluzzii***	-	1	-	0	-	0	-	0	-	0
	***An*. *gambiae s*.*s*.**	0.25	0.13	0.75	0.84	0.13	0.06	0	0	0	0
		N = 2	N = 4	N = 6	N = 27	N = 1	N = 2				
	***An*. *coluzzii-An*. *gam biae s*.*s***	0	0	1	1	0	0	0.17	0	0	-
				N = 6	N = 5			N = 1			
**CRR**	***An*. *arabiensis***	0	1	0.96	0	0	0	0.1	0.27	0	0
			N = 11	N = 22				N = 3	N = 3		
	***An*. *coluzzii***	-	1	-	0	0	0	0	0	0	0
	***An*. *melas***	0	-	1	-	0	-	0	-	-	0

Vgsc- voltage-gated sodium channel. *GSTe2*-glutathione-s-transferase epsilon 2. Ace1-Acetylcholine esterase1. N = number of mosquitoes positive for respective resistance marker.

In *An*. *arabensis* resting indoors in URR, *Vgsc-1014S* frequency was significantly higher (Z = 2.230, P = 0.03) in the indoor- (allele freq. = 0.91, 95%CI: 0.84–0.96) than outdoor-resting (allele freq. = 0.82, 95%CI: 0.76–0.87) mosquitoes. Moreover, *Vgsc-1014S* was the only mutation identified in this species when found resting indoors (allele freq. = 0.96, 95%CI: 0.78–1) in CRR. Whereas in *An*. *coluzzii* in URR, *Vgsc-1014S* mutation was higher in the indoor (allele freq. = 0.25, 95%CI: 0.17–0.36) than outdoor-resting mosquitoes (allele freq. = 0.04, 95%CI: 0.005–1.3) but this was not statistically significant (Z = 0.965, P = 0.33). In LRR, the mutation was found only in indoor-resting mosquitoes (allele freq. = 0.3, 95%CI: 0.19–0.42). In WCR, the mutation was common in *An*. *gambiae s*.*s*. and higher among outdoor- (allele freq. = 0.84, 95%CI: 0.67–0.95) than indoor-resting (allele freq. = 0.75, 95%CI: 0.35–0.97) mosquitoes, with no significant difference (Z = 0.510, P = 0.61).

*Vgsc-1014F* was almost fixed in most mosquitoes, except *An*. *arabiensis*. It was also more common in the outdoor- than indoor-resting mosquitoes. Specifically in URR, the mutation was found to be significantly higher (Z = 2.956, P = 0.003) in outdoor-resting (allele freq. = 0.92, 95%CI: 0.81–0.98) than the indoor-resting *An*. *coluzzi* population (allele freq. = 0.74, 95%CI: 0.63–0.82). Likewise, in the hybrid population of *An*. *coluzzi* and *An*. *gambiae s*.*s*., the mutation was fixed and higher in the outdoor-resting (allele freq. = 1, 95%CI: 0.74–1) than indoor-resting (allele freq. = 0.93, 95%CI: 0.80–1) mosquitoes but not statistically significant (Z = 1.027, P = 0.3). The mutation was similarly fixed in both the indoor (allele freq. = 1, 95%CI: 0.96–1) and outdoor (allele freq. = 0.99, 95%CI: 0.95–1) *An*. *gambiae s*.*s*. populations. Although in WCR, *Vgsc-1014F* was more frequent in *An*. *gambiae s*.*s*. resting indoors (allele freq. = 0.25, 95%CI: 0.03–0.65) than those outdoors (allele freq. = 0.13, 95%CI: 0.04–0.29), the difference was not statistically significant (Z = 0.688, P = 0.49). Whereas in LRR, where only mosquitoes resting indoors were caught, this mutation was most common in *An*. *coluzzii* (allele freq. = 0.66, 95%CI: 0.81–0.98).

*Vgsc-1575Y* and *GSTe2-114T* were found mostly in URR and were more frequent in outdoor-resting mosquitoes. The mutations were almost fixed in *An*. *gambiae s*.*s*. regardless of resting place (allele freq. = 0.96–1, 95% CI: 0.92–1.2). In *An*. *coluzzii*, these mutations were significantly higher (*Vgsc-1575Y*: Z = 3.343, P = 0.001. *GSTe2-114T*: Z = 1.948, P = 0.05) in those resting outdoors (allele freq: 0.9, 95% CI: 0.79–0.97) than in their indoor-resting counterpart (allele freqs: *Vgsc-1575Y* = 0.68, 95% CI: 0.57–0.77. *GSTe2-114T* = 0.78, 95% CI: 0.68–0.86). *An*. *coluzzii* -*An*. *gambiae s*.*s*. hybrids with higher and fixed *Vgsc-1575Y* mutation were equally found resting outdoors (allele freq. = 1, 95% CI: 0.74–1) while those found resting indoors were carrying only the *GSTe2-114T* mutation (allele freq. = 0.87, 95% CI: 0.60–0.98).

The carbamate and organophosphate resistance marker, acetylcholine esterase (*Ace*)*-119S* was detected only in 8 (4 indoor and 4 outdoor) *An*. *gambiae s*.*s*. and in one hybrid specimen in URR.

### Sporozoite infection rate

*Plasmodium falciparum* sporozoites were detected in 11 out of 844 mosquitoes (Table 2), representing a 1.3% (95% CI: 0.5–2%) infection rate. All the infected mosquitoes were caught in URR, of which six were resting indoors and five resting outdoors. Outdoor-resting *An*. *arabiensis* were mostly infected (36%, 4/11), followed by indoor-resting *An*. *gambiae s*.*s*. (27%, 3/11) and indoor-resting *An*. *arabiensis* (18%, 2/11). One each of outdoor-resting *An*. *coluzzii* and *An*. *coluzzii*-A*n*. *gambiae s*.*s*. hybrid were also infected.

**Table 2:** Sporozoite positivity rate in the eleven vector species that were infected based on their resting locations.

	*An*. *arabiensis* Proportion (n)	*An*. *coluzzii* Proportion (n)	*An*. *gambiae s*.*s*. Proportion (n)	*An*. *coluzzii-An*. *gambiae s*.*s*. proportion (n)
Indoor	0.18 (2)	0.09 (1)	0.27 (3)	0
Outdoor	0.36 (4)	0	0	0.09 (1)

n = number of mosquitoes positive for sporozoite detection. Proportion = the number positive per species divided by overall positive (11).

### Host blood meal preference

Host blood meal origin was determined in 251 randomly selected engorged mosquito abdomens. Overall, animal and human blood meal indices were higher for indoor- than outdoor-resting mosquitoes (Table 3). In all vector species, most blood meal (91%) were from animal origin. Indoor-resting *An*. *coluzzii* had the highest preference for human blood while indoor-resting *An*. *arabiensis* had most preference for animal blood. Regardless of their resting location, all vector species preferred cow and donkey blood meal compared to other animals. Most vectors rarely fed on chicken and horse.

**Table 3:** Human and animal blood meal preferences of the indoor and outdoor-resting vector species in combined study sites.

	*An*. *arabiensis*		*An*. *coluzzii*		*An*. *gambiae s*.*s*.		*An*. *coluzzii-An*. *gambiae s*.*s*.	
	Indoor(n)	Outdoor(n)	Indoor(n)	Outdoor(n)	Indoor(n)	Outdoor(n)	Indoor(n)	Outdoor(n)
**Human**	0.01(3)	0.004(1)	0.03(7)	0	0.008(2)	0.008(2)	0	0.004(1)
**Cow**	0.09(22)	0.02(6)	0.06(14)	0.03(8)	0.09(22)	0.08(21)	0.03(7)	0.02(4)
**Chicken**	0	0	0	0	0	0.004(1)	0	0
**Dog**	0.008(2)	0.004(1)	0	0.004(1)	0	0.004(1)	0	0
**Donkey**	0.11(27)	0.12(31)	0.06(14)	0.04(11)	0.02(6)	0.03(8)	0.004(1)	0.008(2)
**Goat**	0.02(4)	0.008(2)	0.008(2)	0.008(2)	0.004(1)	0.004(1)	0.004(1)	0
**Horse**	0.004(1)	0	0	0	0	0	0	0
**Human + animals**	0.004(1)	0.004(1)	0.01(3)	0	0.008(2)	0	0	0
**Mixed animals**	0.008(2)	0	0	0.004(1)	0	0.004(1)	0	0
**HBI**	2	0.8	4	0	1	0.8	0	0.4
**Animal blood indices**	23	16	12	9	12	13	4	2

Proportion (number). HBI = Human blood index.

## Discussion

Insecticide resistance is currently widespread among malaria vectors in The Gambia [11, 14], resulting in insecticide rotation for IRS. Currently, actellic, an organophosphate insecticide is being used, whilst LLINs impregnated with deltamethrin and permethrin as recommended by WHO are distributed widely [32]. This study assessed how such vector interventions have influenced the feeding and resting behaviour, as well as malaria transmission dynamics of the vector population. *Anopheles arabiensis* showed a marked preference for outdoor resting and *An*. *coluzzii* and *An*. *melas* for indoor resting. *An*. *gambiae s*.*s*. and *An*. *coluzzii-An*. *gambiae s*.*s*. hybrid populations were found mostly resting outdoors, but with no statistical difference from those resting indoors. Moreover, local vectors had a marked preference for animal blood. The overall sporozoite infection rate was low and infectious mosquitoes were mainly outdoor-resting *An*. a*rabiensis*.

In this study, high frequencies of *Vgsc-*1014F, *Vgsc-1575Y* and *GSTe2-114T* were recorded in the malaria vector populations resting indoors and outdoors. These mutations were particularly at saturation in both indoor and outdoor-resting *An*. *gambiae s*.*s*. populations in URR, as well as in most of the *An*. *coluzzii*-*An*. *gambiae s*.*s*. hybrid samples analyzed. This magnitude of the DDT and pyrethroid-associated resistance mutations [23, 24, 27] recorded in the vector populations is worrying. These might have been as a result of extensive coverage of IRS and LLINs across The Gambia [7, 8]. These findings suggest that effectiveness of LLINs may be compromised in the local vector populations as pyrethroids remain the only public health-approved insecticide for LLINs [32]. Selection for resistance could have resulted from the extensive use of pyrethroids for vector control [7, 8] and pest control in agriculture [33, 34] in this region. Moreover, the level of resistance found in the *An*. *coluzzii*-*An*. *gambiae s*.*s*. hybrid population suggests an extensive gene flow between *An*. *gambiae* and *An*. *coluzzii* [35–37] in these settings. These observations are a concern for a possible setback to the ongoing anti-vector efforts; thus deserve close monitoring.

A possible influence of genotypic resistance on the resting behaviour of the vector populations [38], was observed from this study. A higher proportion of *An*. *arabiensis* that was found resting indoors harboured the *Vgsc-1014S* mutation whereas *An*. *coluzzii* exhibited an outdoor-resting behavior when all mutation markers except *Vgsc-1014S* were higher in the outdoor than indoor population. *Anopheles gambiae s*.*s*. with extremely high mutations were also found resting both indoors and outdoors. These indicate that resistance could be driving *An*. *coluzzii* and *An*. *gambiae s*.*s*. from their usual indoor resting to outdoor resting behaviour [4, 39, 40], a trait that could promote residual transmission [5, 17, 40]. Indeed, indoor resting behaviour demonstrated in the resistant vectors despite the presence of IRS and LLINs suggests that resistance could be protecting these vectors against the effect of insecticides used indoors [41, 42]. This has been previously reported from studies in Kenya [43, 44], where *An*. *gambiae s*.*s* and *An*. *arabiensis* that had higher frequencies of *Vgsc-1014F* and *Vgsc-1014S* respectively, were predominantly found resting indoors. Similarly, association between genotypic resistance and outdoor-resting behavior was earlier found in *An*. *coluzzii* [45, 46]. Any resistance-driven resting behavior in these vectors could further limit the success of anti-vector interventions currently being scaled-up in these settings [47, 48].

The majority of the vector populations analyzed had a marked preference for animal than human blood meal. *Anopheles arabiensis* demonstrated predominant preference for animal blood meal than other vector species. This is not surprising as *An*. *arabiensis* is known to be highly zoophilic [49, 50]. However, the proportion of vectors resting indoors and that have taken a blood meal either from human and animal source, could be a concern for the effectiveness of vector control measures. As animals were found only in outdoor locations in the study sites, this finding shows that the vectors took blood meal from animals outdoors and later went indoors to rest regardless of the presence of IRS and LLINs, indicating that these vectors are resistant to the insecticides being used [51]. Furthermore, the observed choice of animal blood by majority of the vectors could lead to increase in vector population that may eventually resort to biting humans in the long run and become difficult to control. Notably, alternative vector control methods such as treatment of animals with endectocides [52] and larval source management [53] could be promising tools that could be adopted by the Gambia National Malaria Control Program.

This study recorded an overall low sporozoite rate in the vector populations. Given the current low malaria prevalence in The Gambia [7], a low sporozoite rate is expected. This may reflect the impact of the scaled-up in IRS and LLINs program in the study sites which seems to successfully limit mosquito access to human blood meal indoors and consequently reducing transmission, as previously reported [54, 55]. Indeed, the study sites benefitted from improved housing projects conducted in country which aimed at reducing mosquito survival as well as malaria transmission [56].

The composition of the vector species was consistent with previous studies in the Gambia where the most abundant vector was *An*. *arabiensis*, followed by *An*. *gambiae s*.*s*. and, *An*. *coluzzii* along with their hybrids [11, 14, 15]. Low density of *An*. *melas* found was as a result of our choice of villages in the West, which were not located in the coastal regions where this species breeds in salty water [57]. Remarkably, predominance of *An*. *arabiensis* could be as a result of its outdoor-resting preference to avoid insecticide used in IRS and LLINs [58, 59]. This leaves the highly anthropophilic and endophilic species more exposed to vector interventions, possibly leading to relative advantage that maintains the exophilic population and malaria transmission [59].

## Conclusion

This study observed high levels of resistance mutations in the local vectors that could be influencing their resting behaviour. As The Gambia is in earnest preparation for pre-elimination phase of malaria, the magnitude of resistance mutations observed in the vectors in this study suggests that vectors could pose great challenges for their control using the present control paradigm. Therefore, use of larval source management as a complementary vector control measure is highly recommended.

## Supporting information

S1 File Overall data including separate worksheets for genotypic resistance and species genotypes; host blood meal and sporozoite positivity data. (XLSX)
